# Genotypic diversity, a survival strategy for the apicomplexan parasite *Theileria parva*

**DOI:** 10.1016/j.vetpar.2009.09.025

**Published:** 2010-02-10

**Authors:** F. Katzer, D. Ngugi, A.R. Walker, D.J. McKeever

**Affiliations:** a Moredun Research Institute, Pentlands Science Park, Bush Loan, Penicuik, Midlothian EH26 0PZ, United Kingdom; b Royal Veterinary College, Hawkshead Lane, North Mymms, Hatfield, Hertfordshire AL9 7TA, United Kingdom; c Royal (Dick) School of Veterinary Studies, Easter Bush Veterinary Centre, University of Edinburgh, Midlothian EH25 9RG, United Kingdom

**Keywords:** *Theileria*, Diversity, Genetic markers, Selection

## Abstract

The tick-borne protozoan parasite *Theileria parva* causes East Coast fever (ECF), a severe lymphoproliferative disease of cattle that is a major constraint to the improvement of livestock in eastern, central and southern Africa. Studies in cattle experimentally infected with *T. parva* have shown that the protective cytotoxic T lymphocyte (CTL) response is tightly focused, with individual animals recognizing only one or two dominant antigens, the identity of which varies with MHC class I phenotype. It is well known that cross-protection between *T. parva* stocks is limited, but precise evaluation of genetic diversity in field populations of the parasite has been hampered by a lack of molecular markers spanning the genome. A recently described panel of satellite markers has provided evidence for substantial genotypic diversity and recombination but does not provide cover for large segments of the genome. To address this deficiency, we undertook to identify additional polymorphic markers covering these regions and we report herein 42 newly identified PCR-RFLP markers distributed across the 4 *T. parva* chromosomes, as well as 19 new satellite markers for chromosomes 1 and 2. This brings the total number of available polymorphic markers to 141 for the 8.5 Mb genome. We have used these markers to characterise existing parasite stabilates and have also shown that passage of the parasite through naïve cattle and ticks can lead to substantial changes of parasite populations in resulting stabilates. These markers have also been used to show that passage of mixed parasites through an immunised calf results in the removal of the immunising genotype from the parasite population produced by ticks fed on this animal.

## 1 Introduction

The protozoan parasite *Theileria parva* is transmitted by *Rhipicephalus* ticks and causes an often fatal lymphoproliferative disease of cattle known as East Coast fever (ECF). In keeping with the distribution of its principal vectors, *Rhipicephalus appendiculatus* and *R. zambeziensis*, the disease is prevalent in eastern, central and southern Africa (Lawrence et al., 1994), where an estimated 24 million cattle are at risk of infection. Economic losses due to ECF are substantial, as evidenced by a 1989 analysis that placed them at US$168 (Mukhebi et al., 1992). Efforts to control ECF are largely based on the use of acaricides to prevent infestation with infected ticks, but this approach is increasingly being compromised by the emergence of acaricide resistance in the vector tick populations. Although drugs are available to treat the disease, these are expensive and require an early diagnosis to be effective. It is also possible to immunise cattle against *T. parva* by inoculation with live parasites in combination with long acting formulations of oxytetracycline. This so-called infection and treatment method (Radley et al., 1975a,b) is effective, but its uptake has been hampered by cold chain difficulties and concerns that vaccine strains might establish in resident tick populations and mix with local parasite genotypes (Oura et al., 2007).

The parasite has a complex life cycle (Mehlhorn and Schein, 1984), involving obligate developmental stages in mammalian and vector hosts. Cattle become infected by inoculation of sporozoite forms in the tick saliva. These invade lymphocytes and differentiate to multinucleate schizonts, which drive the cell into a state of continuous proliferation and divide with it, ensuring transmission of infection to each daughter cell. In a proportion of infected cells, schizonts undergo further differentiation to uninucleate merozoites; these are released from the dying cell and invade erythrocytes, where they develop into tick-infective piroplasm forms. Upon ingestion by a feeding tick, these are released into the gut lumen and give rise to macro and micro gametes, which undergo syngamy to form diploid zygotes. After invading gut epithelial cells, zygotes undergo reduction division to yield kinete forms, which access the hemocoel and migrate to the salivary gland, where they invade cells of type III acini. The parasite then undergoes a process of sporogony to produce cattle-infective sporozoites. The parasite therefore adopts a strategy whereby expansion is accomplished through asexual division, with an exponential phase in the case of the schizont, while genetic exchange is accommodated through a sexual phase in the tick (Neitz, 1957).

Early observations of limited cross-protection among field isolates of *T. parva* prompted a strong interest in the diversity of the parasite. This led to the generation of monoclonal antibodies that could distinguish between different parasite isolates (Minami et al., 1983; Conrad et al., 1989). The advent of DNA-based technologies gave rise to the development of a series of new markers that could distinguish multiple genotypes simultaneously. The first of these were Southern blot-based and used combinations of restriction enzymes and probes for the small subunit ribosomal RNA gene as well as probes for multi-copy genes from the TpR and sub telomeric loci (Bishop et al., 1993). These markers showed extensive polymorphism between isolates but the results of mixed infections were more difficult to interpret. PCR-RFLPs of the 18S ribosomal RNA locus, Polymorphic Immunodominant Molecule (PIM) PCR amplicon size polymorphisms and PIM sequence analysis were also used to distinguish different isolates and stocks (Bazarusanga et al., 2007; Geysen et al., 2004). However, the availability of the *T. parva* genome allowed the identification of satellite sequences comprising multiple nucleotide repeats, which, because of varying numbers of repeats, give rise to alleles with distinct PCR amplicon sizes. Satellite markers have provided insights into the genetic relationship between *T. parva* populations (Oura et al., 2003; Katzer et al., 2006) and the population dynamics and sub-structuring of field isolates (Oura et al., 2005; Odongo et al., 2006). They have also been applied to elucidate the impact of immune selection on the parasite (Katzer et al., 2007) and the risks associated with the infection and treatment method (Oura et al., 2004, 2007). At a more practical level, the source of a recent outbreak of ECF on the Comoros Islands was traced to an infection and treatment vaccine used in cattle exported from Tanzania using microsatellite profiling (De Deken et al., 2007).

Although many polymorphic markers now exist for the distinction of different *T. parva* genotypes, large regions of its genome remain for which no markers are available. Effective evaluation of recombination rates and genotypic variation in response to selection pressure requires a genome-wide set of markers with good coverage. We therefore undertook to develop additional satellite and PCR-RFLP markers to get a better genome-wide coverage.

## 2 Materials and methods

### 2.1 Parasite material

The study focused on the Marikebuni stock of *T. parva*, which was initially isolated in 1981 in the Kilifi District in Kenya (Minami et al., 1983). It underwent three cattle–tick passages at the International Livestock Research Institute (ILRI) in Nairobi to yield ILRI St3014 (Morzaria et al., 1995). This stabilate was passaged again at the National Veterinary Research Centre, NVRC, in Kenya and infected ticks were sent to the Centre for Tropical Veterinary Medicine, University of Edinburgh. These were used to generate stabilate CTVM St70, which has since been passaged twice to generate stabilates CTVM St72 and CTVM St96. The history of the *T. parva* Muguga stabilate CTVM-ST80 is less well defined. This isolate was maintained for many years by serial cattle–tick passage and underwent several further cattle–tick passages after importation into the UK.

### 2.2 Animal immunisation, challenge and stabilate production

A Friesian calf was infected by subcutaneous inoculation with 1 × 10^7^ autologous cells infected with the 72-01 genotype of *T. parva* Marikebuni (see below). The animal showed only mild clinical signs and recovered without treatment. The immune status of the calf was confirmed by detection of *in vitro* MHC-restricted CTL activity against the immunising cell line as described by Goddeeris and Morrison (1988). The animal was challenged two months after the initial infection with a lethal dose of the parent stabilate CTVM St72 by subcutaneous inoculation above the right prescapular lymph node. Progress of infection was followed by monitoring rectal temperature and examining lymph node biopsy and blood smears for the presence of schizonts and piroplasms respectively. Unfed nymphal *R. appendiculatus* ticks were applied to the ears of the calf from day 10 of challenge to allow passage of the break-through infection and production of a daughter stabilate. The level of infection in the resulting tick batch was assessed by examination of salivary glands from a representative sample as described by Walker et al. (1979) and stabilate CTVM St105 was prepared from it as described by Brown (1987).

### 2.3 Generation of parasite clones and lysis for PCR

Parasite clones for genotyping were generated by limiting dilution cloning of PBMCs infected *in vitro* with *T. parva* sporozoite stabilates or lymph node aspirates obtained from an infected animal as described by Rocchi et al. (2008). Parasite clones were grown in 96 well plates, harvested and lysed, by incubation for 10 h at 56 °C in culture medium containing 50 μg/ml Proteinase K. PCR reactions were conducted directly on the lysates after heating to 90 °C for 10 min to inactivate Proteinase K.

### 2.4 Polymorphic markers and genotyping

(i) *PCR-RFLPs*: Comparative analysis of the genomic sequences of *T. annulata* and *T. parva* (http://www.sanger.ac.uk and http://www.tigr.org respectively) was undertaken to identify genes that are polymorphic between the two species. Polymorphic coding regions that differed in size between the species were selected and primer pairs flanking the most variable segments were designed to amplify products ranging from 600 bp to 1800 bp. PCR amplicons generated from *T. parva* Muguga and Marikebuni clones using these primers were digested with a panel of 10 frequent cutting restriction enzymes (AluI, DpnII, HaeIII, HhaI, HinfI, MseI, MspI, RsaI, TaqI and Tsp509I) to identify RFLPs. Details of the polymorphic PCR-RFLP markers are shown in Table 1.
(ii) *Satellite markers*: The satellite markers ms1–ms11, MS1–MS46, MS221a, MS221b, MS312, MS717 and MS817 have been published previously by Oura et al. (2003) and markers MS47–MS59 have been described by Katzer et al. (2006). The repeat finder program (Benson, 1999) was used as described by Oura et al. (2003) to identify 35 additional satellite loci on chromosomes 1 and 2 for testing.
(iii) *Size polymorphisms*: In addition to the 4 previously described genes that show PCR size polymorphisms among *T. parva* isolates (Katzer et al., 2006), one new marker, TP02-0895, was identified on chromosome 2 with size variants detectable by PCR (forward primer gcctgtcaagagtaccttaatgcc, reverse primer gaccgcttggctgacctggacc).
(iv) *PCR conditions and genotyping*: PCR conditions used in the study were essentially as described by Oura et al. (2003), except that the number of cycles was increased to 40, Bioline (UK) taq polymerase was used and a custom made 10× PCR buffer (45 mM Tris–HCl (pH 8.8), 11 mM (NH_4_)_2_SO_4_, 4.5 mM MgCl_2_, 0.113 mg/ml BSA, 4.4 μM EDTA, 1.0 mM each of dATP, dCTP, dGTP and dTTP) was purchased from ABgene (UK). PCR products were separated on 2% Metasieve agarose (Flowgen, UK), visualised with ethidium bromide and photographed using a UV light box (BioRad, UK).

## 3 Results

### 3.1 Genetic tools to study *T. parva*

**Fig. 1:**
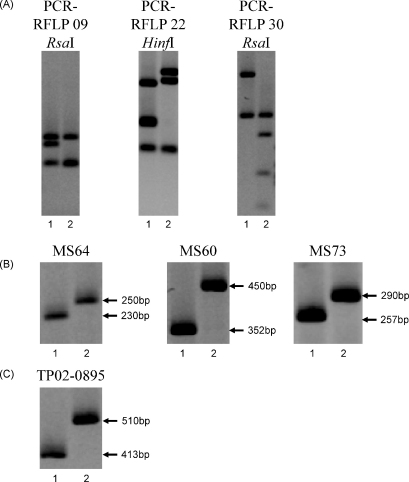
Examples of polymorphic markers identified in this study. (A) PCR-RFLP markers with corresponding restriction enzyme. (B) Newly identified satellite markers. (C) TP02-0895 a newly discovered gene with amplicon size polymorphism. Lane 1, Muguga clone 273; lane 2, Marikebuni clone 3219.

**Fig. 2:**
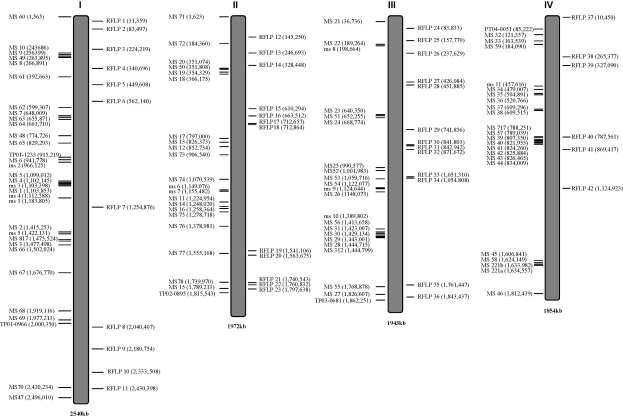
Graphical illustration of the locations of polymorphic markers in the *T. parva* genome. Chromosomes 1–4 are denoted by I–IV. Genes with amplicon size polymorphism and satellite marker loci are shown on the left hand side of each chromosomes, while PCR-RFLP locations are shown on the right. Sizes of individual chromosomes are identified below.

Comparison of the *T. annulata* and *T. parva* genomes led to the identification of 105 open reading frames that exhibited sequence variation or gaps in the sequence alignment and these were used to design *T. parva*-specific primers flanking the polymorphic regions. Digestion of PCR products, obtained from *T. parva* Muguga and Marikebuni clones with these primers, with a panel of 10 restriction enzymes led to the identification of 42 PCR-RFLPs, 3 of which are shown in Fig. 1. A list of all PCR-RFLPS, along with their primer sequences, relevant restriction enzyme and location in the *T. parva* genome are shown in Table 1. Large sections of chromosomes 1 and 2 were devoid of satellite markers and these regions were therefore re-examined using the repeat finder program (Benson, 1999) in an attempt to identify more markers in those regions. As a result, 36 further primer pairs were designed and tested with DNA from *T. parva* Muguga and Marikebuni clones and 19 of these markers were found to be polymorphic, 3 of which are shown in Fig. 1. The names, primer sequences and Muguga amplicon sizes of these polymorphic satellite markers are shown in Table 2. An illustration of the locations of all new and previously identified satellite markers and newly identified PCR-RFLP markers are shown in Fig. 2. The figure shows the location of all 94 polymorphic satellite markers, 42 PCR-RFLPs and the 5 genes which exhibit amplicon size polymorphisms. Of these markers, 43, 34, 35 and 29 markers locate on chromosome 1, 2, 3 and 4, respectively. Of the 29 markers on chromosome 4, 12 are tightly clustered into two groups, separated by a stretch of over 772 kb that lacks any polymorphic satellite markers. This stretch contains only 2 polymorphic PCR-RFLP markers, leaving chromosome 4 with the largest stretch for which polymorphic markers are unavailable.

### 3.2 Parasite population structure changes during cattle–tick passage

**Fig. 3:**
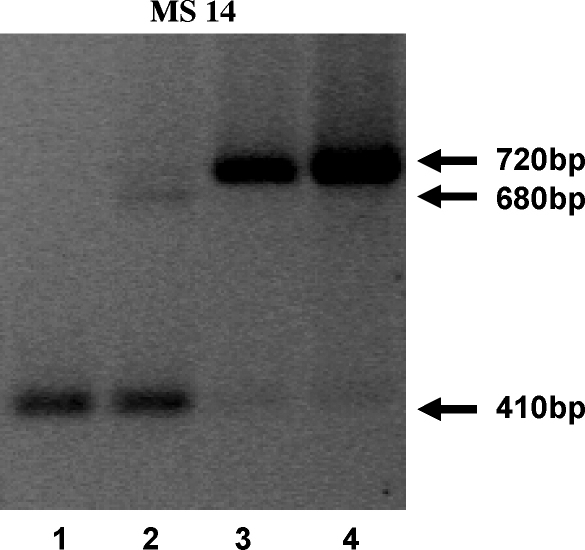
Satellite analysis of whole genomic DNA extracted from four successive generations of the *T. parva* Marikebuni isolate using marker MS14. Lane 1, ILRI St3014; lane 2, CTVM St70; lane 3, CTVM St72; lane 4, CTVM St96. The arrows mark amplicon sizes in base pairs.

PCR analysis of DNA extracted from *T. parva* Marikebuni stabilates derived from four serial cattle–tick passages (ILRI St3014–CTVM St70–CTVM St72–CTVM St96) with a panel of satellite markers revealed that individual passages can lead to marked changes in population structure. Representative results using the MS14 satellite marker are illustrated in Fig. 3 and show that, while ILRI 3014 is dominated by one allele of this marker, its passage to CTVM 70 was associated with the emergence of at least two additional alleles. After further passage to CTVM St72, one of these new alleles has become dominant, while the original ILRI 3014 allele is indiscernible. This genotypic profile was retained after an additional passage of the stock to yield CTVM St96. Multi locus genotyping (MLG) of clones obtained from CTVM St72 and St96 with a panel of 69 satellite markers, as well as 5 PCR size polymorphisms and 3 PCR-RFLPs, confirmed that both stabilates are dominated by the same genotype (72-01). Of 287 clones obtained from stabilate CTVM St72, 218 (76%) carried the 72-01 genotype, while most of the remaining 69 clones were singletons. Similarly, the 72-01 genotype accounted for 76.2% of clones analysed from CTVM 96 and the frequency of 72-01 alleles carried by the remainder was higher than that observed in CTVM St72.

### 3.3 Evidence of immune selection

**Fig. 4:**
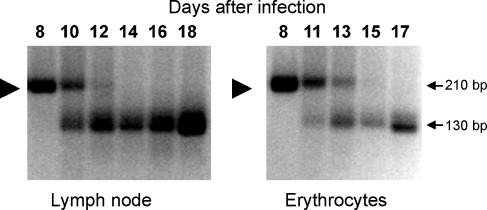
Clearance of the immunising parasite genotype 72-01 in lymph node and erythrocyte compartments on the indicated days after challenge with stabilate CTVM St72 as revealed by PCR amplification with satellite marker MS27. The arrowheads denote the allele carried by the immunising genotype 72-01. The arrows mark amplicon sizes in base pairs.

The impact of the bovine immune response on the progression of *T. parva* infection was studied by challenging a calf immunised against the 72-01 genotype with the parent stabilate CTVM St72. The immune status of the calf was confirmed prior to challenge by detection of parasite-specific CTL *in vitro*. The animal developed mild clinical signs following challenge, with transient fever and emergence of both schizonts and piroplasms, and recovered without treatment. Ticks fed on the animal following challenge exhibited low infection rates but were used to generate a working stabilate, CTVM St105. Parasite clones were obtained from lymph node aspirates collected on days 9 (*n* = 34) and 14 (*n* = 27) of the challenge infection and from cloned parasitized cell lines derived by *in vitro* infection with CTVM St105 (*n* = 75). MLG analysis of the ex vivo-derived clones with a subset of 69 polymorphic markers, including 5 genes with PCR size polymorphisms, revealed only 7 occurrences of the 72-01 genotype at day 9. The genotype was not detected among the day 14 clones or those generated *in vitro* using the CTVM St105 stabilate. A significant reduction in the prevalence of the 72-01 genotype was also apparent in DNA amplified from serial lymph node aspirates and erythrocyte theileriosis fractions collected during the challenge infection. This is illustrated in Fig. 4 using the polymorphic marker MS27, which is representative of several markers for both non-coding and coding regions (Fig. 4). In addition, the 72-10 alleles of four expressed genes (TP01-0966, TP01-1233, TP03-0681, TP04-0051) were absent from over 82% of the parasite clones analysed from the breakthrough stabilate CTVM-St105 (data not shown).

## 4 Discussion

We describe an expanded set of molecular markers for the study of population diversity in *T. parva* parasites. The panel now comprises 141 PCR based markers that are distributed across the genome as depicted in Fig. 2, which shows revised position for previously published satellite markers on chromosome 3 (Oura et al., 2003). The markers are not evenly distributed, but instead often cluster together in certain regions of the four chromosomes. Attempts to identify markers in gaps have met with only limited success, suggesting that the regions that lack polymorphic markers may represent regions in which the *T. parva* genome is conserved across different isolates. Alternatively, they may simply reflect conservation between the Muguga and Marikebuni isolates in these regions of the genome. It is therefore possible that testing markers that failed to reveal polymorphisms in this study might do so if tested on a broader range of isolates. This might reveal more markers for chromosome 4 and provide a sufficient marker density to support an approach for strain specific antigen identification similar to that adopted by Martinelli et al. (2005) in their genetic approach to mapping targets of strain specific immunity in malaria.

These genetic tools have allowed us to evaluate parasite diversity in both distinct and related *T. parva* stabilates. Analysis of the *T. parva* Muguga stabilate CTVM St80 has shown that the stabilate is almost clonal (data not shown); out of 48 clones analysed 45 were identical and only 4 multi locus genotypes were observed. These were distinguished by differences at only 3 satellite loci. In the case of one locus, one of the observed alleles was novel, while the other 2 had been seen before in Marikebuni clones. The novel allele may have arisen through spontaneous mutation within the Muguga stabilate. Alternatively, this and the other 2 alleles may reflect true diversity that has survived many cattle to tick passages and remain the only examples of polymorphism left from the original Muguga isolate.

The observation of a total of 70 genotypes within the CTVM St72 was surprising as analysis of bulk stabilate DNA with individual markers suggested the presence of a dominant genotype along with, possibly, one or two others. This level of diversity, coupled with the substantial changes observed after a single cattle–tick passage, has important implications for the design of experiments that rely on *T. parva* stabilates. Workers in the past have assumed that the genetic composition of heterogeneous stabilates is relatively stable.

This has important implications for maintenance of the “Muguga cocktail”, which forms the basis of infection and treatment immunisation in eastern Africa. Comprising three heterogeneous stocks – Muguga, Kiambu 5 and Serengeti transformed – there is clearly a danger that repeated passage will result in changes in the antigenic composition of the cocktail. The molecular typing reagents described in this paper provide a valuable tool to characterise the individual components of the “Muguga cocktail” and, possibly, to identify which genotype or genotype-combinations are essential to confer protection against natural field challenge. The identification of the protective components will be essential for quality assurance of the next generation of the “Muguga cocktail”.

The underlying cause of the observed changes in parasite composition of successive *T. parva* Marikebuni stabilates remains unclear. The history of these stabilates has shown that three tick lines were used to propagate the parasite during the course of these passages, which occurred at ILRI, NVRC Muguga and the CTVM. Hence, ILRI ticks were used to generate ILRI St3014, CTVM St70 arose from the tick colony at the NVRC and CTVM St72 and St96 were generated with ticks from the CTVM colony. Another possible reason for these changes is that distinct cattle breeds were used at each stage—Boran cattle were used at ILRI, the NVRC used a Boran-Friesian cross and Friesian calves were used at the CTVM. Alternatively, the emergence of dominant genotypes or changes of genotype composition may simply be stochastic, representing a chance event in which a given parasite genotype manages to establish initially a more vigorous infection in the bovine host and thereby subsequently dominates the infection in the tick host.

Immunisation with the 72-01 genotype followed by challenge with CTVM St72, in which the 72-01 genotype accounts for 76% of the parasite population, has shown that while the bovine immune response can clear the immunising parasite genotype during the challenge infection, other genotypes can persist and be transmitted to feeding ticks. This is in line with the reported tight focus of the CTL response (see McKeever, 2006). This has very important implications for the sustained use of a given infection and treatment vaccine in the same geographical area. It may lead to the selection of local parasite strains, which are antigenicly distinct from the vaccine components, which, in time would result in failure of the vaccine.

## Conflict of interest statement

None of the authors (F. Katzer, D. Ngugi, A.R. Walker, D.J. McKeever) has a financial or personal relationship with other people or organisations that could inappropriately influence or bias the paper entitled “Genotypic diversity, a survival strategy for the apicomplexan parasite *Theileria parva*”.

## Figures and Tables

**Table 1:** PCR-RFLP marker loci identified in this study^a^.

Marker Name	Forward Primer	Reverse Primer	Restriction Enzyme	Chromosome No.	Position	Amplicon Size
PCR-RFLP 01	acagggatgattctggtaattttt	tggktggcataagtamtctgtgat	RsaI	1	31359	698
PCR-RFLP 02	amcgcaraatatyaaaacagaact	tttttgacctacttaaatcatttgaca	RsaI	1	83497	928
PCR-RFLP 03	ccakgagaaccttaacactgmctc	agtcaagtttcrtcttcgtttcct	MspI	1	224219	682
PCR-RFLP 04	ttgttaataccacsccaattatca	aggktcyaaatactttcccaaaa	TaqI	1	340696	694
PCR-RFLP 05	ygacgaagataatatggayatgga	ctccatacagtgctctcgttacat	RsaI	1	449608	1555
PCR-RFLP 06	cacaaggagagttatttgcgtct	tcctyttcatcatcttctcraaca	TaqI	1	562140	642
PCR-RFLP 07	attgacgttctcaaaattggtgat	tccatttttgaagccatttttatt	HinfI	1	1254876	625
PCR-RFLP 08	tgttctttttatttggactcttttcc	ttggtcttctgctgtgtaagaagt	HinfI	1	2040407	1564
PCR-RFLP 09	gataagttgttacgcacatgggtt	atttggatcgctaactagtctgc	RsaI	1	2180754	923
PCR-RFLP 10	tacttggtgcaatttctagtctcg	ggcgagttgtggtaaatctca	RsaI	1	2333508	1435
PCR-RFLP 11	accgggtttcagaaagtttttaat	gcgaggaaattgatgagaagtagt	TaqI	1	2430398	1138
PCR-RFLP 12	ctttgagaaatggctcaatgagta	tgcagaggagagtgttaggatara	RsaI	2	145250	1202
PCR-RFLP 13	ctaggagttaacccaggaacmag	ctgatttggacttcgattcytttt	TaqI	2	246693	1644
PCR-RFLP 14	ttcgcaaatttaccaaagttttta	tcatccaagggttaattttcctaa	MspI	2	328448	1566
PCR-RFLP 15	gtgacacatttaaccccaactatg	cgtgtttaacctccatcctcttta	MspI	2	610294	992
PCR-RFLP 16	ttggaccatggatttaaagagttt	gataaattcaagcaaatcaaccaa	MseI	2	663512	848
PCR-RFLP 17	ggtaaacctttaggtgtgtttgga	tgccaaataatcctccagtagtag	AluI	2	712657	1021
PCR-RFLP 18	gagagtgattttggagaaggctac	gactcaaccttcttcgctactctc	MspI	2	712864	1715
PCR-RFLP 19	gaaaaatgccgaaaaataaagg	atcgaaatccgactcctcttg	MseI	2	1541106	695
PCR-RFLP 20	ttcattgttaccaccaaatcttc	cgacactttccacatcgttacata	HaeIII	2	1563675	1224
PCR-RFLP 21	ggatatgataaagattggtataaatgg	tcacagttaaatcaacagctgctac	TaqI	2	1740543	742
PCR-RFLP 22	taaaccacaaggagctcttcctac	aacgacgccttaaactttctatac	HinfI	2	1760832	1401
PCR-RFLP 23	gaayggattwagatttgatgtggt	atttmccagattccattraataaa	HhaI	2	1797638	847
PCR-RFLP 24	tctgaacgacttgcagtatgacta	gactccaacacaacgaattca	HhaI	3	83833	695
PCR-RFLP 25	ggtatttaagggtagaattggagg	tttccataaaggatcaatattctcaa	MseI	3	157770	781
PCR-RFLP 26	ttctgatcccctgatacaattttt	ttatgttaccgcaatcaccaatag	HinfI	3	237629	1426
PCR-RFLP 27	tgaagcctgtcaagttgcttta	tgcgttacatacacttcccttg	AluI	3	426084	1783
PCR-RFLP 28	aagaacactcagttatgaaggctgt	ccctcatcttaccactcaatttct	HaeIII	3	451885	841
PCR-RFLP 29	ccagctgtatactcacttgttgct	aacttgttttcctttggcttagg	AluI	3	741856	968
PCR-RFLP 30	acggtttatgacaagtctgtacca	tcgaacgagtgttttaactttttg	RsaI	3	841803	1198
PCR-RFLP 31	tgagttatttgaggaaggatttgag	ttttaaagagtcccaagtgttcaa	HhaI	3	842942	766
PCR-RFLP 32	cacgtatgtatcccaagtatccac	gaggatttgagaacccagttacc	MseI	3	871672	1100
PCR-RFLP 33	tgcttaaaggctcagttatcacaa	acaaattcgggtatgtttttgaa	RsaI	3	1051310	973
PCR-RFLP 34	gaaaaactgctcaaactccgttat	aaagtactcgtggtctggagtctg	AluI	3	1054808	1119
PCR-RFLP 35	atctcaaatggctttgctaaactt	gcaaataatattgcagataccagaa	MseI	3	1761447	908
PCR-RFLP 36	tatatacacttcckgtwrtcggta	gttcatcgtttttcccataaaca	MseI	3	1843437	1469
PCR-RFLP 37	ttcatattattcggatctgtgaga	tctccgtcacatactacttgttca	HaeIII	4	10450	1555
PCR-RFLP 38	cagtctcaacaattgggacagata	gtaacttctccttcatttccttgg	RsaI	4	265377	1123
PCR-RFLP 39	tccataggtattctcgaaggtct	gtgttcctatctcaccctccaac	MseI	4	327090	777
PCR-RFLP 40	gacgctagactacgatgaaatgaa	gtgcactctcaaacgcctaakmat	Tsp509I	4	787561	630
PCR-RFLP 41	gggaacacaaaccaagcaag	atctgcctcagtgccttcat	AluI	4	869417	922
PCR-RFLP 42	cgttgtaggcttaatgatgaactt	catcattgattttagcggtgaat	RsaI	4	1124923	1142

a The amplicon sizes shown are those found in the *T. parva* Muguga clone 3308 (http://www.tigr.org).

**Table 2:** New satellite marker loci identified in this study^a^.

Marker Name	Forward Primer	Reverse Primer	Chromosome No.	Position	Amplicon Size
MS60	aatctgagggtcaaaggatt	tcaatcaacatgttatcagga	1	1565	352
MS61	gaagagggtactgaagctga	aggatcagtagctggagttg	1	392663	248
MS62	gcaaaatcgaactaccacat	cgctctagcctctgtaacac	1	599307	271
MS63	tcattccatcggatctttat	tggtaaaacttcgtaaaaagg	1	655871	263
MS64	acatccttaggcacaacatc	gctgcctcatgtacaggtat	1	661710	230
MS65	tgctcaattcccaatacaa	tccatttccttaaccacatc	1	829293	251
MS66	ctaccactatcaccggtagc	catcagcgttacttgcatc	1	1502024	266
MS67	ctcgtttagaaaagccagaa	gtctctttatcagcagcttca	1	1676770	245
MS68	tcacatcgggtaacaagaa	tatttatcgaccccaaactg	1	1919116	469
MS69	atgtgtacagcaatcaacga	catctgaagactcctccaaa	1	1977213	245
MS70	actcatttgcaccgtatctt	aactctggaatctcaaccaa	1	2420234	227
MS71	aggtggttaggaccattagg	gttgttgattcagaggttcc	2	1623	252
MS72	ttcacaatgaattctgagga	aaatttcattgcttgatttga	2	184360	230
MS73	tccttgtggttcaagtaaaac	caaaacctcacttcaccttt	2	906540	257
MS74	gactctggaggggaaaga	gtgttaaccacgggaaaag	2	1070539	246
MS75	ccaccccgtctactatatca	ttcacacaacgcttcttaaa	2	1278718	244
MS76	ggtgtgcacttaagcagttt	tgaaggacttttcacacaaat	2	1378981	253
MS77	ggtaaccaacaaccacattt	tgcttatgaactcaatcatctc	2	1555168	270
MS78	caaccaatctactcccaact	tggatattccaatcgattattag	2	1739970	361

a The amplicon sizes shown are those found in the *T. parva* Muguga clone 3308 (http://www.tigr.org).
